# Evaluation of recombinant baculovirus clearance during rAAV production in Sf9 cells using a newly developed fluorescent-TCID_50_ assay

**DOI:** 10.3389/fmed.2024.1302648

**Published:** 2024-01-22

**Authors:** Ningguang Luo, Xiangqun Chen, Jinzhong Li, Derrick Huynh, Ying Li, Li Ou, Shengjiang Liu

**Affiliations:** Avirmax CMC Inc., Hayward, CA, United States

**Keywords:** recombinant adeno-associated virus (rAAV), recombinant baculovirus (rBV), fluorescent-TCID_50_ (F-TCID_50_) assay, Sf9 cells, gene therapy

## Abstract

**Introduction:**

Recombinant adeno-associated virus (rAAV) vectors provide a safe and efficient means for *in vivo* gene delivery, although its large-scale production remains challenging. Featuring high manufacturing speed, flexible product design, and inherent safety and scalability, the baculovirus/Sf9 cell system offers a practical solution to the production of rAAV vectors in large quantities and high purity. Nonetheless, removal and inactivation of recombinant baculoviruses during downstream purification of rAAV vectors remain critical prior to clinical application.

**Methods:**

The present study utilized a newly developed fluorescent-TCID_50_ (F-TCID_50_) assay to determine the infectious titer of recombinant baculovirus (rBV) stock after baculovirus removal and inactivation, and to evaluate the impact of various reagents and solutions on rBV infectivity.

**Results and discussion:**

The results showed that a combination of sodium lauryl sulfate (SLS) and Triton X-100 lysis, AAVx affinity chromatography, low pH hold (pH3.0), CsCl ultracentrifugation, and NFR filtration led to effective removal and/or inactivation of recombinant baculoviruses, and achieved a log reduction value (LRV) of more than 18.9 for the entire AAV purification process. In summary, this study establishes a standard protocol for downstream baculovirus removal and inactivation and a reliable F-TCID_50_ assay to detect rBV infectivity, which can be widely applied in AAV manufacturing using the baculovirus system.

## 1 Introduction

Gene therapy typically involves packaging a gene within a vector to facilitate its entry into target cells and provide therapeutic benefits to the affected individuals (1). While retrovirus (an enveloped, ssRNA virus) vectors are mostly utilized to rectify a default gene in blood cells recovered from the patient followed by re-infusion via *ex vivo* gene therapy, adeno-associated virus (AAV) vectors are preferred for diseases that affect specific organs, such as the brain, spinal cord, or liver, via systemic or local gene delivery (2). Between 1998 and 2022, there were 255 clinical trials using AAV delivery globally (3), with six regulatory approvals (4).

To date, several types of recombinant adeno-associated virus (rAAV) vector production systems, including those using human embryonic kidney cells (HEK293) (5) and insect *Spodoptera frugiperda* (Sf9) cells (6), have been developed. Due to their helper functions that are necessary for efficient AAV DNA replication, baculovirus (an enveloped dsDNA virus) has been adopted for AAV production since 2002 (6). Additional improvements in the AAV capsid subunit (VP1) have been made to enhance rAAV transducibility (7), and another VP1-rich system based on Sf9-AAV production, which utilizes baculovirus for rAAV packaging during the upstream process in the Sf9 cell culture system, has also been described (8). Furthermore, the contaminated *S. frugiperda* rhabdovirus (Sf-rhabdovirus, ssRNA virus), which persistently infects Sf9 cell lines, was reported (9, 10). However, Sf-rhabdovirus does not enter or replicate in human cell lines and is, thus, unlikely to be a risk for gene therapy applications (11). Integration of intron splicing-mediated expression of AAV Rep and Cap genes into the design of the transgene expression cassette enables large-scale production of AAV vectors in insect cells (12). The first Sf9-derived gene therapy product (alipogene tiparvovec, sold under the brand name Glybera, for reversing lipoprotein lipase deficiency or LPLD) gained regulatory authorization for the market in 2012 (13), and the other regulatory approvals are Roctavian^®^ and Hemgenix for hemophilia A and B, respectively. The use of Sf9 cells in rAVV production has been continuously increasing from approximately 6% before 2007 to 20% in 2022 (3). Three (43%) of the seven rAAV gene therapy products that have been approved so far (an additional approval from Sarepta Therapeutics for Duchenne muscular dystrophy in 2023) are manufactured using the Sf9 system. These products indicate that the Sf9 system produced rAAV vectors with a pretty high successful approval rate, considering the relatively low percentage (18%) of AAV clinical trials using the Sf9 system.

The virus infectivity titer is usually expressed as the median tissue culture infectious dose (TCID_50_), which is sufficient to cause a distinguishable cytopathic effect (CPE) in 50% of wells containing an indicator cell culture. In the Sf9 cell culture, the cells attach to the surface of culture plates and have round shapes. However, unlike the visible CPE observed in the cell-based virus assays for detecting mammalian viruses by the TCID_50_ assay, baculovirus-induced morphological changes in the Sf9 cells, such as increased cell size and decreased viability, are difficult to identify under a regular light microscope. Therefore, an accurate determination of the baculovirus assay endpoint is often challenging. To overcome this detection barrier, the Sf9 cells were inoculated at 28°C for 6–8 days with a recombinant baculovirus (rBV) carrying a green fluorescent protein (GFP) gene for easy visualization of green foci in the infected cells under a fluorescent microscope (14).

**Figure 1:**
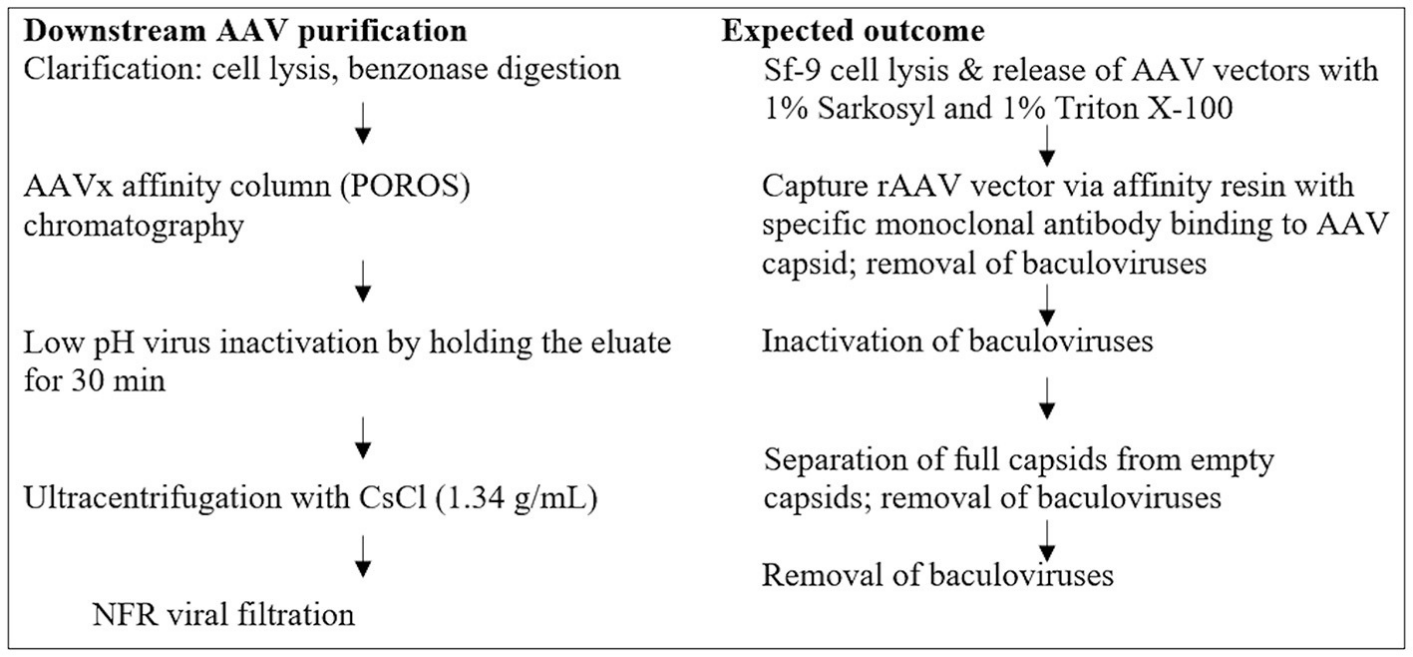
Steps for rBV removal and/or inactivation during downstream AAV purification.

In unprocessed harvest bulk containing a high titer of rBV, Sf9 cell lines have been shown to persistently carry Sf rhabdovirus (Sf-RV), an enveloped, negative sense, and single-stranded RNA (-ssRNA) virus. Apparently, Sf-RV is insect-specific and non-infectious to mammalian and human cell lines. However, utilizing an Sf-RV-containing cell line for producing biologics provokes a viral safety risk and additional complexity in clearing the virus during production. To eliminate potential viral safety risks associated with the rAAV products, a combination of viral inactivation and/or removal steps has been developed and incorporated into the AAV manufacturing platform. These steps include rBV inactivation by sodium lauryl sulfate (Sarkosyl, SLS) and Triton X-100 during cell lysis, rBV removal by AAVx affinity chromatography, rBV inactivation by low pH hold, and rBV removal by CsCl ultracentrifugation and viral filtration. Each step has been assessed to ensure the functionality, purity, and safety of the final rAAV product. In this study, a fluorescent-TCID_50_ (F-TCID_50_) assay was developed to monitor rBV removal and/or inactivation during each step in the AAV purification platform, as shown in Figure 1. The results showed that the implementation of multiple AAV purification steps led to effective inactivation and/or removal of rBV and achieved a log reduction value (LRV) of more than 18.9 for the entire AAV purification process.

## 2 Materials and methods

### 2.1 Sf9 cell culture maintenance

The Sf9 cells (Expression Systems) were cultured in ESF AF media (Expression Systems, Davis, CA, USA) containing 100 units/ml penicillin and 100 μg/ml streptomycin (Fisher Scientific, CA, USA) in Corning bottles at 28°C with gentle shaking at 160 rpm. Once cells were expanded to 1 ± 0.2 × 10^7^ cells/ml, they were split 1:2 to 1:8 with fresh medium and continuously cultured for maintenance.

### 2.2 Generation of rBV-GFP stock with high titers and development of the F-TCID_50_ assay

The rBV-GFP, a recombinant baculovirus carrying a GFP cassette under the control of the baculovirus p10 promoter, was generated by using a Bac-to-Bac baculovirus expression system (Thermo Fischer Scientific, Fremont, CA, USA). To obtain an rBV stock with high titers, the rBV-GFP (P0) was amplified by infecting 400 ml of the Sf9 cell culture at a multiplicity of infection (MOI) of 0.1 in a 1,000-ml Corning bottle at 28°C with shaking at 180 rpm for 3 days. After removal of the cell pellets by centrifugation at 2,000 rpm for 5 min, the baculovirus was concentrated by ultracentrifugation at 24,000 rpm using a Beckman 45Ti rotor for 2 h at 4°C with 25% and 60% sucrose gradient cushion made with a virus stock buffer (25 mM Tris–HCl pH 7.3, 100 mM NaCl, 5 ml EDTA, and 1 mg/ml BSA). At the end of the centrifugation, the virus band from each tube was carefully drawn and combined. After measuring the titer using quantitative polymerase chain reaction (qPCR), the rBV-GFP pool was aliquoted and stored at −80°C.

A fluorescent-TCID_50_ (F-TCID_50_) assay was developed to determine the infectious titer of rBV-GFP stock. Briefly, Sf9 cells were seeded in 96-well plates at a density of 4 × 10^5^ cells/ml and 50 μl/well. A series of dilutions of rBV-GFP stock made with ESF AF medium from 10^−1^ to 10^−11^ were prepared, and 50 μl of each dilution was added to each well of the plate seeded with Sf9 cells, from low to high, with the same dilution on each column. Fresh medium was added to the wells in the last column as the negative control. The cells were incubated at 28°C for 6–8 days prior to the assessment for GFP expression using fluorescent microscopy. A well was counted as positive if one or more green foci, indicative of rBV-GFP infection, were detected. If no green foci were detected, the well was considered negative. The infectious titer of rBV-GFP stock was calculated using the Spearman–Karber equation. For a more accurate assessment of the infectivity of baculovirus in rBV-GFP-spiked samples during AAV purification, serial dilutions at 1:3.2 (instead of 1:10) were utilized in the F-TCID_50_ assay.

### 2.3 Toxicity of reagents and solutions to Sf9 cells

Two 96-well plates, with seeded Sf9 cells at 4 × 10^5^ cells/mL and 50 μL/well, were prepared for each test sample under examination. Each reagent or solution to be tested was diluted with an ESF AF medium from 1:3.2^1^ to 1:3.2^11^, with or without the rBV-GFP stock spiked at 1:10,000 (v/v), and 50 μL of each dilution was added to each well of the two plates, one with and the other without the spiked rBV-GFP. After incubation at 28°C for 6–8 days, the cells without added rBV-GFP were examined under light microscopy, and the cells with spiked rBV-GFP were visualized under fluorescent microscopy as described in the F-TCID_50_ assay.

### 2.4 Interference of reagents and solutions with rBV infectivity in Sf9 cells

A volume of 10 ml of each diluted reagent or solution tested for cell toxicity was prepared using a fresh ESF AF medium. The dilution factor was determined based on the result from the toxicity assay (Table 1) as described above. In general, the first dilution was the minimum dilution of the reagent or solution without observable cell toxicity, indicated by morphological changes in the cells. Serial dilutions at 1:3.2 were then prepared up to three times. For example, if a reagent showed no toxicity at a 1:10 (1:3.2^2^) dilution and above, the first dilution would be 1:10 (1:3.2^2^), followed by 1:32 (1:3.2^3^) and 1:100 (1:3.2^4^) dilutions. The rBV-GFP stock was spiked into 10 ml of fresh medium or each prepared dilution at 1% (v/v). The F-TCID_50_ assay was performed using a fresh medium or each corresponding dilution using 1:3.2 serial dilutions.

**Table 1:** Toxicity of reagents and solutions on Sf9 cells.

**Sample**	**Minimum dilution factor (fold)**	**Corresponding infectious titer or possible LoD (log_10_(TCID_50_)/ml)**
ESF AF (positive control)	3.2	1.3
1% (w/v) SLS	3.2^7^	4.3
1% (v/v) Triton X-100	3.2^7^	4.3
Native lysate^*^	3.2	1.3
Processed lysate^**^	3.2^7^	4.3
AAVx eluate pH 3.0	3.2^3^	2.3
Neutralized AAVx eluate, pH 7.5	3.2	1.3
CsCl (1.34 g/ml) in PBS	3.2^4^	2.8
AVMX formulation buffer	3.2	1.3
AVMX formulation buffer with AAV	3.2	1.3

^*^Sf9 cell lysate generated by freeze-thaw at −80°C three times. ^**^Sf9 cell lysate generated by lysis buffer containing both SLS and Triton X-100.

### 2.5 Kinetic study of rBV inactivation during lysis and low pH treatments

rBV-GFP was spiked (1:20) into reagents or solutions adopted for cell lysis and/or low pH hold during AAV manufacturing, including 1% (w/v) SLS, 1% (v/v) Triton X-100, mock lysate containing both 1% (w/v) SLS and 1% (v/v) Triton X-100, and AAVx elution buffer with pH adjusted to 3. Briefly, a 25-μl rBV-GFP stock was spiked into 475 μl of the above reagents or solutions and vortexed to mix; 40 μl out of the 500-μl virus-spiked samples was immediately collected and quenched by adding to sterile 15-ml tubes containing 3,960 μl (1:100) ESF AF medium immediately (T0) or after 5 min (T5), 30 min (T30), 60 min (T60), and 120 min (T120), respectively. The F-TCID_50_ assay was performed immediately after medium quench at each time point to evaluate rBV-GFP infectious titers in the virus-containing solutions using 1:3.2 serial dilutions. Cells lysed by three cycles of a freeze-thaw and ESF AF medium were used as the cell lysate control and the positive control.

### 2.6 Buffer exchange using Amicon-4 centrifugal filter units

Amicon-4 centrifugal filter units were used for buffer exchange to remove toxic reagents in virus-spiked samples and increase the sensitivity of the assay. Briefly, 4 ml of diluted virus-spiked samples containing different detergents was concentrated to approximately 1 ml by spinning at 7,500 rpm for 4–5 min. The concentrated sample volume was brought up to 4 ml using a fresh ESF AF medium, mixed, and spun two more times. The F-TCID_50_ assay was then performed as described above using 1:3.2 serial dilutions.

### 2.7 Evaluation of rBV removal by AAVx affinity chromatography, CsCl ultracentrifugation, and viral filtration

rBV-GFP was spiked into bulk samples before each mock purification step, including AAVx affinity chromatography, CsCl ultracentrifugation, and NFR viral filtration. The F-TCID50 assay was performed before and after the aforementioned purification step, and the LRV was calculated to determine the rBV removal efficiency of each step.

## 3 Results

### 3.1 Toxicity of reagents and solutions to Sf9 cells

**Figure 2:**
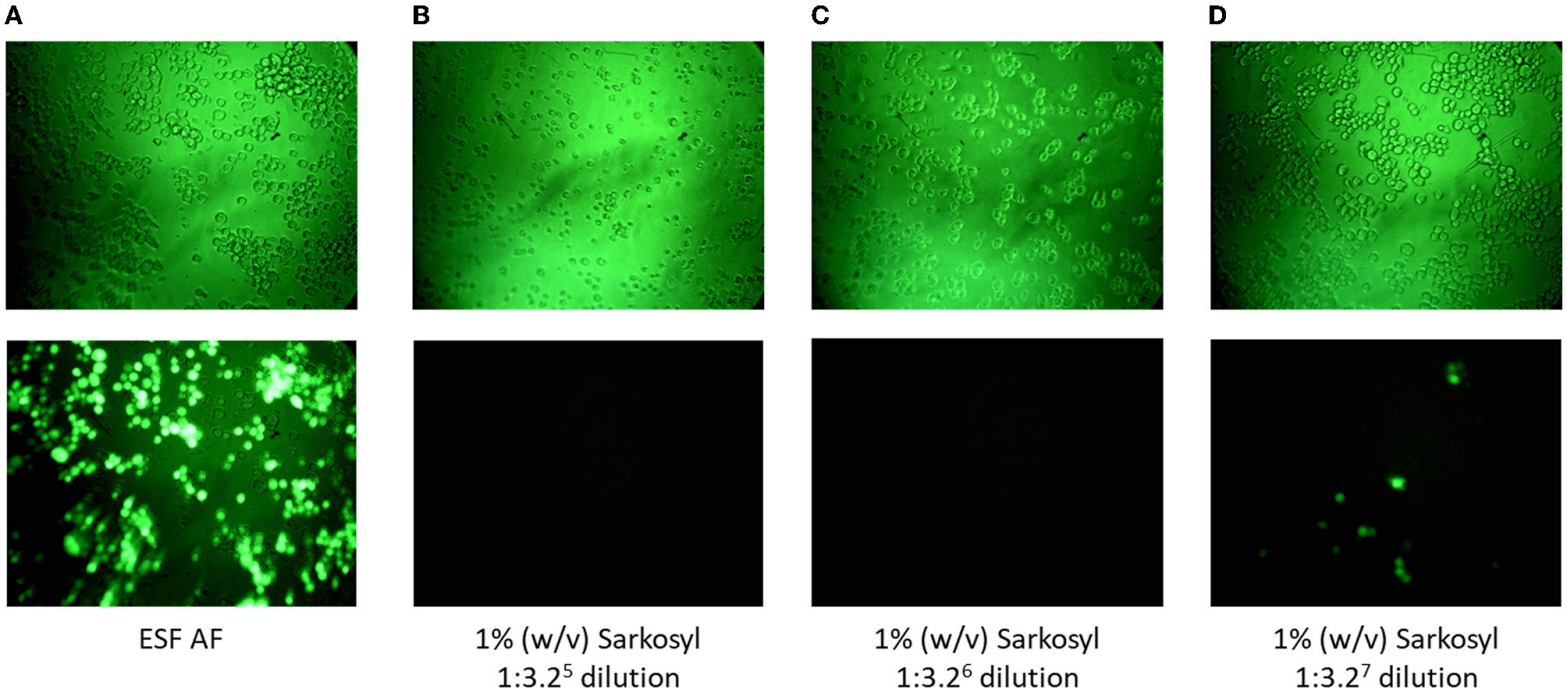
Cell toxicity was demonstrated by morphological changes and a lack of green foci in the presence of spiked rBV-GFP. **(A)** Cells cultured in ESF AF medium. **(B–D)** Cells cultured with ESF AF medium in the presence of 1% (w/v) SLS at different dilutions of 1:3.25 **(B)**, 1:3.26 **(C)**, and 1:3.27 **(D)**. Top panels, cells under 20 × light microscopy and bottom panels, cells under 20 × fluorescent microscopy after incubation with rBV-GFP as described in the Materials and Methods section.

Since the absence of rBV infectivity and/or the presence of cell toxicity might lead to negative results (no green foci) in the F-TCID_50_ assay, cell toxicity due to each test reagent or solution used during AAV purification was examined to identify the potential causes of negative results. As summarized in Table 1, different concentrations of SLS, Triton X-100, low pH, or CsCl were toxic to Sf9 cells to different extents, manifesting as morphological changes in cells (Figures 2B, C). No green foci were detected in the corresponding wells containing spiked rBV-GFP (Figure 2). The minimum dilution factor of each reagent or solution was determined once the cell morphological changes were significantly reduced or disappeared and green foci started to appear (Figure 2D). As shown in Figure 2, compared to cells in the medium only, those in the presence of 1% (w/v) Sarkosly at a dilution of 1:10^6^ or below significantly shrank and had no detectable green foci in the corresponding GFP spiked wells, while those in the presence of 1% (w/v) SLS at a dilution of 1:10^7^ appeared much closer to normal, with some detectable green foci in the corresponding GFP spiked wells. Therefore, the 1% (w/v) SLS was considered non-toxic after dilutions at 10^7^-fold or above. Similarly, among other test reagents and solutions listed in Table 1, Triton X-100 at 1% (v/v) and processed lysate containing both SLS at 1% (w/v) and Triton at 1% (v/v) were considered non-toxic after dilutions at 10^7^-fold or above; CsCl at 1.34 g/ml was considered non-toxic after dilutions at 10^4^-fold or above, and AAVx eluate at low pH (3.0) was considered non-toxic after dilutions at 10^3^-fold or above; all other samples tested, including neutralized AAVx eluate (pH 7.5) and AAV formulation buffer, were considered non-toxic after 1:3.2 dilution with a ESF AF medium. An AAV sample spiked in the AVMX formulation buffer at 2 × 10^13^ vg/ml to simulate the AAV purification process displayed no detectable cell toxicity. The infectious titers of rBV at the corresponding non-toxic dilution levels were calculated based on the Spearman–Karber equation.

To increase the assay sensitivity for monitoring rBV inactivation by toxic reagents or solutions, such as detergents and CsCl in PBS, buffer exchange using the ESF AF medium was performed before each F-TCID_50_ assay. The infectious titers of rBV in the ESF AF medium were similar before and after buffer exchange, indicating no significant rBV loss during the process. On the other hand, the cell toxicity of the reagents was significantly reduced after buffer exchange, and the loss of green loci was largely due to rBV-GFP inactivation and not due to the toxicity of the reagents (Table 2). The difference in minimum dilution factors was approximately 30–100 (3.2^3^-3.2^4^) folds before and after buffer exchange, indicating a 1.5–2 increase in the limit of detection (LoD) in the F-TCID_50_ assay (Tables 1, 2).

**Table 2:** Toxicity of reagents and solutions on Sf9 cells after buffer exchange.

**Sample**	**Minimum dilution factor (fold)**	**Corresponding infectious titer or possible LoD [log_10_(TCID_50_)/ml]**
ESF AF (positive control)	3.2	1.3
1% (w/v) SLS after buffer exchange	3.2^3^	2.3
1% (v/v) Triton X-100 after buffer exchange	3.2^4^	2.8
Processed lysate^*^ after buffer exchange	3.2^4^	2.8
CsCl in PBS after buffer exchange	3.2	1.3

^*^Sf9 cell lysate generated by lysis buffer containing both SLS and Triton X-100.

### 3.2 Interference of rBV infectivity on Sf9 cells by reagents and solutions

Samples showing no signs of Sf9 cell cytotoxicity may exhibit viral interference, which impacts the ability of the virus to establish infection in host cells. This type of viral interference was observed during the cell toxicity evaluation. Despite the relatively healthy appearance of Sf9 cells at the minimum dilutions, green loci in the corresponding wells, containing spiked rBV-GFP, were significantly reduced or eliminated in some cases (Figure 2). Given that the neutralized AAVx eluate with dilutions at 1:3.2 and above was regarded as non-toxic (Table 1), dilutions of the neutralized AAVx eluate were prepared with the ESF AF medium at 1:3.2, 1:3.2^2^ (1:10), and 1:3.2^3^ (1:32), and the infectivity of spiked rBV-GFP in those dilutions was compared to the control in the ESF AF medium (Table 3). The results indicated that there was no interference from AAVx eluate pH 7.5 at dilutions of 1:3.2^2^ (1:10) and above. Therefore, the 1:3.2^2^ (1:10) dilution of AAVx eluate pH 7.5 was regarded as a non-interfering dilution. Similarly, the 1:10,000 (1:3.2^8^) dilution of 1% (w/v) SLS, 1% (v/v) Triton X-100, processed lysate, the 1:100 (1:3.2^4^) dilution of AAVx eluate pH 3.0, the 1:1,000 (1:3.2^6^) dilution of CsCl in PBS at 1.34 g/ml, and the 1:3.2 dilution of AVMX formulation buffer were non-interfering (Table 3). The calculated corresponding infectious titer in the F-TCID_50_ assay of each reagent and solution at its minimum non-interfering dilution (highlighted in bold in Table 3) was used as LoD for rBV inactivation analysis.

**Table 3:** Interference with rBV infectivity on Sf9 cells by test reagents.

**Dilution**	**Log_10_ (TCID_50_)/ml**	**LRV**	**Corresponding infectious titer [log_10_(TCID_50_)/ml]**
ESF AF (PC)	6.8 ± 0.2	NA	1.3^*^
1% SLS, 1:3.2^7^	5.7 ± 0.2	1.1 ± 0.2	4.3
1% SLS, 1:3.2^8^	6.8 ± 0.4	0.0 ± 0.4	**4.8** ^**^
1% SLS, 1:3.2^9^	6.5 ± 0.2	0.3 ± 0.2	5.3
1% Triton X-100, 1:3.2^7^	5.4 ± 0.3	1.4 ± 0.4	4.3
1% Triton X-100, 1:3.2^8^	6.5 ± 0.3	0.3 ± 0.4	**4.8**
1% Triton X-100, 1:3.2^9^	6.5 ± 0.3	0.3 ± 0.4	5.3
Processed lysate^***^, 1:3.2^7^	5.9 ± 0.2	0.9 ± 0.2	4.3
Processed lysate, 1:3.2^8^	6.5 ± 0.2	0.3 ± 0.2	**4.8**
Processed lysate, 1:3.2^9^	6.8 ± 0.2	0.0 ± 0.2	5.3
AAVx eluate pH 3.0 1:3.2^3^	5.7 ± 0.2	1.1 ± 0.2	2.3
AAVx eluate pH 3.0 1:3.2^4^	6.4 ± 0.3	0.4 ± 0.4	**2.8**
AAVx eluate pH 3.0 1:3.2^5^	6.5 ± 0.3	0.3 ± 0.4	3.3
AAVx eluate pH 7.5 1:3.2	4.0 ± 0.2	2.8 ± 0.2	1.3
AAVx eluate pH 7.5 1:3.2^2^	6.9 ± 0.2	00.±0.2	**1.8**
AAVx eluate pH 7.5 1:3.2^3^	6.8 ± 0.2	0.0 ± 0.2	2.3
1.34 g/cc CsCl 1:3.2^4^	5.5 ± 0.3	1.3 ± 0.4	2.8
1.34 g/cc CsCl 1:3.2^5^	6.0 ± 0.3	0.8 ± 0.4	3.3
1.34 g/cc CsCl 1:3.2^6^	6.7 ± 0.2	0.1 ± 0.2	**3.8**
AVMX formulation buffer 1:3.2	6.9 ± 0.2	0.0 ± 0.2	**1.3**
AVMX formulation buffer 1:3.2^2^	6.9 ± 0.1	0.0 ± 0.2	1.8
AVMX formulation buffer 1:3.2^3^	6.7 ± 0.2	0.1 ± 0.2	2.3

^*^The minimum dilution factor was 3.2 in the F-TCID_50_ assay. ^**^The LoD of each tested reagent or solution was highlighted in bold. ^***^V432AG Sf9 cell lysate generated by lysis buffer containing both SLS and Triton X-100.

### 3.3 Assessment of rBV inactivation by 1% (w/v) SLS and/or 1% (w/v) Triton X-100

The rBV-GFP stock was spiked at 20% (v/v) into Sf9 cells containing medium only (control cells), 1% (w/v) SLS, 1% (v/v) Triton X-100, or Sf9 cell lysate with both detergents. The rBV-GFP spiked test samples were quenched by a 1:100 dilution with the medium at time points of 0 min (T0), 5 min (T5), 10 min (T10), 30 min (T30), 60 min (T60), and 120 min (T120). The F-TCID_50_ assay was performed immediately at each time point, and the results showed that significant rBV inactivation was observed at T0, and prolonged detergent treatment did not significantly increase rBV inactivation (data not shown). Therefore, T0 was selected for buffer exchange using Amicon ultra-4 centrifugal filter units before the F-TCID_50_ analysis. A medium without spiked rBV was included as the negative control (NC) to assess cell toxicity, and the medium with spiked rBV was included as the positive control (PC) to determine the infectivity of spiked rBV. The control cells were native lysates of V432AG Sf9 cells, an engineered Sf9 cell line used for AAV manufacturing, and served as a control to evaluate the impact of cell lysate without any detergent treatment on the infectivity of spiked rBV. No significant differences in rBV infectious titer were observed in control cells before and after buffer exchange, indicating that the infectious titer of spiked rBV-GFP was not affected by control cells or buffer exchange. After treatment with each detergent or detergents containing lysate, the infectivity of spiked rBV was significantly reduced from 7.1 ± 0.2 to ≤ 2.3 (1% SLS), 3.5 ± 0.3 (Triton X-100), or ≤ 2.8 (lysate), as summarized in Table 4. The corresponding LRV was ≥4.8 ± 0.2 in the SLS-treated sample, ≥4.3 ± 0.2 in the lysate sample, or 3.6 ± 0.4 in the Triton X-100-treated samples. The results indicated that rBV was more efficiently inactivated by SLS than Triton X-100. The observation of slightly higher rBV infectivity in the lysate treatment (containing both Salkosyl and Triton X-100) than that in the SLS group was due to more efficient buffer exchange in the SLS-treated sample.

**Table 4:** Inactivation of rBV-GFP by detergents.

**Sample/infectivity log_10_(TCID_50_)/ml**	Control cells^*^	**1% (w/v) Sarkosyl**	**1% (w/v) Triton X-100**	Processed lysate^**^
Negative control	≤ 1.3 ± 0.0	≤ 2.3 ± 0.0	≤ 2.8 ± 0.0	≤ 2.8 ± 0.0
Positive control	7.1 ± 0.2	7.1 ± 0.2	7.1 ± 0.2	7.1 ± 0.2
After treatment	7.2 ± 0.2	≤ 2.3 ± 0.0	3.5 ± 0.3	≤ 2.8 ± 0.0
LRV	−0.1 ± 0.4	≥4.8 ± 0.2	3.6 ± 0.4	≥4.3 ± 0.2

^*^V432AG Sf-9 cell lysate generated by freeze-thaw at −80°C three times. ^**^V432AG Sf-9 cell lysate generated by lysis buffer containing both Sarkosyl and Triton X-100.

### 3.4 Assessment of rBV removal by AAVx affinity chromatography

V432AG Sf9 cells cultured in bioreactors were mechanically lysed by sonication without detergents or benzonase prior to depth filtration and 0.2 μm filtration. The rBV-GFP stock was spiked at 2% (v/v) into the processed samples immediately before loading onto an AAVx affinity column (AAVx load). AAVx affinity column eluate was collected, immediately neutralized to pH 7.5, and 0.22-μm filtered (AAVx eluate) before analysis using the F-TCID_50_ assay. The toxicity and interference assays showed that neutralized AAVx affinity column eluate (pH 7.5) was non-cytotoxic at 1:3.2 and non-interfering at 1:3.2^2^ (1:10) dilutions (Tables 1, 3). Thus, the LoD for AAVx affinity chromatography was 1.8 Log_10_(TCID_50_)/ml. The rBV infectivity was reduced from 6.1 ± 0.3 (AAVx load) to ≤ 1.8 (AAVx eluate), with an LRV of ≥4.3 ± 0.3 after the sample was purified using AAVx affinity chromatography (Table 5).

**Table 5:** rBV removal by AAVx affinity chromatography.

**Sample**	**Vol (ml)**	**Log_10_(F-TCID_50_)/ml**	**F-TCID_50_/ml**	**Total F-TCID_50_**	**Log_10_(F-TCID_50_)**	**LRV**
AAVx Load	500	4.5 ± 0.3^*^	3.16 × 10^4^	1.58 × 10^7^	7.2	
AAVx Eluate	12	≤ 1.3^*^	5.01	60.1	1.8	5.4

^*^AAVx Load and AAVx Eluate were 1:50 diluted with ESF AF before the assay.

### 3.5 Assessment of rBV inactivation by AAVx column eluate adjusted to pH 3.0

The rBV-GFP stock was spiked at 20% (v/v) into an AAVx eluate with pH adjusted to 3.0 and incubated at room temperature in the virus inactivation kinetic study. Aliquots of virus-spiked samples were collected at 0 min (T = 0), 5 min (T = 5), 10 min (T = 10), 30 min (T = 30), 60 min (T = 60), and 120 min (T = 120) and quenched by 1:100 dilution with the medium immediately after each collection. The F-TCID_50_ assay was performed immediately after the medium quench at each time point. A significant reduction in rBV-GFP infectivity was detected immediately after exposure to low pH, and the reduced infectivity was maintained with low pH treatment for up to 120 min (Table 6). Medium only and AAVx eluate at pH 3.0 without spiked rBV served as negative controls to determine the LoD of the F-TCID_50_ assay. The rBV-spiked medium was collected at T = 0 (initial positive control) and T = 120 min (end positive control), and the result indicated that rBV was not inactivated by incubation in the medium at room temperature for up to 2 h. Buffer exchange to remove cell toxicity caused by low pH was unnecessary since detectable green loci were observed in the assay plate, indicating that cell toxicity from low pH did not change the assay results. LRV after low pH treatment for 30 min was approximately 2.9 ± 0.4. Therefore, more than 99.7% of spiked baculovirus was inactivated by a low pH hold for 30 min, a standard AAV purification procedure. Interestingly, 99.7% of rBV-GFP was inactivated immediately at pH 3.0, and an increased incubation time did not significantly increase the inactivation efficiency.

**Table 6:** Baculovirus inactivation by low-pH incubation.

**Sample/infectivity log_10_(TCID_50_)/ml**	**Control cell**	**AAVx eluate pH 3.0**
Negative control	≤ 1.3	≤ 2.3
Initial positive control	7.4 ± 0.2	7.4 ± 0.2
End positive control	7.4 ± 0.2	7.4 ± 0.2
T0	7.2 ± 0.3	4.8 ± 0.2
T5	7.0 ± 0.2	4.5 ± 0.2
T10	7.4 ± 0.2	4.3 ± 0.2
T30	7.2 ± 0.3	4.5 ± 0.3
T60	7.3 ± 0.2	4.3 ± 0.3
T120	7.2 ± 0.3	4.4 ± 0.2
LRV	0.2 ± 0.4	2.9 ± 0.4

### 3.6 Assessment of rBV removal and inactivation by ultracentrifugation

The rBV-GFP stock was spiked at 2% (v/v) into sterile neutralized AAVx affinity column eluate containing 1.34 g/ml CsCl (UC load) immediately before ultracentrifugation at 65,000 rpm and 15°C for 20 h using a 70Ti rotor, a comparable purification step to AAV manufacturing. rBV removal and inactivation after centrifugation were evaluated using the F-TCID_50_ assay (Table 7). ESF-AF medium spiked with rBV-GFP served as the positive control. After ultracentrifugation for 20 h, the rBV infectivity was reduced from 7.2 (UC-load) to 5.1 (AAV full band) log_10_(F-TCID_50_), a LRV of 2.1.

**Table 7:** rBV inactivation and removal by ultracentrifugation.

**Sample**	**Vol (ml)**	**Log_10_(F-TCID_50_)/ml**	**F-TCID_50_/ml**	**Total F-TCID_50_**	**Log_10_(F-TCID_50_)**	**LRV from PC**	**LRV from UC-load**
Medium (PC^*^)	12.5	6.3 ± 0.3	2.00 × 10^8^	2.49 × 10^9^	9.4	0	NA
UC-load	12.5	4.1 ± 0.3	1.26 × 10^6^	1.57 × 10^7^	7.2	2.2	0
AAV full^**^	2.5	2.7 ± 0.3	5.01 × 10^4^	1.25 × 10^5^	5.1	4.3	2.1

^*^PC, positive control. ^**^After buffer exchange using Amicon-4 centrifugal filter units.

### 3.7 Assessment of rBV removal by NFR filtration

After ultracentrifugation, the AAV full band was collected and 1:10 diluted with AVMX formulation buffer to bring the AAV titer closer to 2.5 × 10^12^ vg/ml. The rBV-GFP stock was spiked at 2% (v/v) into the ultracentrifugation-processed AAV sample, and an aliquot of the spiked sample was collected and 0.22 μm filtered (NFR-load). The remainder of the virus-spiked sample was immediately loaded onto an NFR filter. During viral filtration, fractions of the filtrate were collected, and an aliquot of each fraction was 0.22 μm filtered prior to the F-TCID_50_ assay. The results showed that the infectivity of the rBV-spiked NFR load was similar to that of the rBV-spiked ESF-AF medium, indicating that no rBV inactivation was observed at this step (Table 8). Compared with the LRV of 2.2 from rBV inactivation during the ultracentrifugation step, the stable rBV activity was probably due to the shorter incubation time and lower concentration of CsCl. AVMX formulation buffer was non-cytotoxic and non-interfering at 1:3.2 dilution, and the LoD was 1.3 log_10_(TCID_50_)/ml (Tables 1, 3). Therefore, LRV was more than 5.1 ± 0.3 after viral filtration by NFR (Table 8). The AAV recovery after NFR filtration was more than 80%, as determined by the QPCR analysis (data not shown). Thus, NFR filtration appeared to be a very effective step in removing rBV with high AAV recovery during AAV manufacturing.

**Table 8:** rBV removal by NFR filtration.

**Sample**	**Infectivity of LoD [log_10_(TCID_50_)/mL]**	**LRV**
ESF AF medium (positive control)	6.2 ± 0.3	NA
Before NFR filtration (load)	6.4 ± 0.3	NA
After NRF filtration fraction	≤ 1.3	≥5.1 ± 0.3

## 4 Discussion

Recombinant adeno-associated virus (rAAV) vectors have proven a safe and efficient tool for gene therapy applications due largely to their superior biosafety rating, low toxicity, a broad range of infectivity (in both dividing and quiescent cells), and stable *in vivo* transgene expression (15, 16). However, large-scale manufacturing of rAAV vectors has been a bottleneck for their widespread adoption in clinical practice. Given that recombinant baculovirus (rBV) demonstrates high manufacturing speed, flexible product design, and inherent safety and scalability, it has the potential to enhance large-scale production of rAAV vectors (17–19). Restricted to specific insects, baculoviruses do not replicate in cells from mammals, birds, fish, plants, or non-target insects and are thus considered biosafe (20). The use of Sf-9 cells, which are derived from ovarian cells of the non-biting insect fall armyworm *Spodoptera frugiperda* and can grow in low-cost and serum-free media, allows efficient production of rBV stock. Indeed, the baculovirus/Sf9 cell system represents one of the most advanced platforms for rAAV manufacturing to date (21).

Notwithstanding its ability to produce high-titer and high-purity rBV, the baculovirus/Sf9 cell system sometimes leaves a low amount of baculovirus DNA contamination that requires removal before clinical application. Furthermore, it is important to remove and inactivate recombinant baculoviruses for maximal therapeutic efficacy during downstream purification of rAAV vectors (22, 23). Toward this goal, the present study developed an F-TCID_50_ assay to determine the infectious titer of rBV-GFP stock after baculovirus removal and inactivation and to evaluate the impact of various reagents and solutions on the rBV infectivity. As summarized in Table 9, a combination of SLS and Triton X-100 lysis, AAVx affinity chromatography, low pH hold (pH 3.0), CsCl ultracentrifugation, and NFR filtration led to effective inactivation and/or removal of recombinant baculoviruses and achieved a LRV of more than 18.9 for the entire AAV purification process. These data provided strong evidence that the multiple AAV purification steps implemented (Supplementary Table) are adequate for rBV removal and/or inactivation.

**Table 9:** Summary of rBV removal and inactivation during AAV manufacturing at AvirmaxCMC.

**Process**	**Type**	**LRV**
Lysis (Sarkosyl and Triton X-100)	Inactivation	≥4.3 ± 0.2
AAVx affinity chromatography	Removal	5.4
Low pH hold (pH 3.0)	Inactivation	2.9 ± 0.4
CsCl ultracentrifugation	Removal and Inactivation	2.1
NFR filtration	Removal	≥5.1 ± 0.3
Total		≥18.9

Although this F-TCID_50_ assay is useful for its cost-effectiveness, convenience, and adaptability to industry settings, it is subject to inherent variability as a cell-based assay. Therefore, a flow cytometry-based assay may be an alternative for more quantitative analyses. Nevertheless, as shown in the results, this F-TCID_50_ assay is sufficiently accurate and reliable, which warrants a broad application. In the traditional TCID_50_ assays, the infected cells/clusters usually show significant cytopathic effects due to the infection, such as changing shapes or being degranulated. However, the infected insect cells with rBV infection, which could enlarge, for example, are difficult to differentiate from normal/non-infected cells under the microscope. In contrast, in the F-TCID_50_ assay, infected cells with green foci (fluorescence) are easy to detect. In addition, residual bacmid or rBV could be measured using qPCR, ddPCR, or NGS. In a recent study, DNA impurities, including baculoviral contamination, were observed through PacBio and Illumina sequencing (19). The F-TCID_50_ assay only examines live rBV-GFP, which can infect cells. The titer of inactivated rBV-GFP may be high, but the F-TCID50 will be negative if rBV is inactivated, for example, by detergent or low pH.

## Data availability statement

The original contributions presented in the study are included in the article/supplementary material, further inquiries can be directed to the corresponding author.

## Ethics statement

Ethical approval was not required for the studies on animals in accordance with the local legislation and institutional requirements because only commercially available established cell lines were used.

## Author contributions

NL: Conceptualization, Data curation, Formal analysis, Investigation, Software, Validation, Visualization, Writing—original draft, Writing—review & editing. XC: Formal analysis, Validation, Writing—review & editing. JL: Formal analysis, Investigation, Validation, Writing—review & editing. DH: Formal analysis, Validation, Writing—review & editing. YL: Formal analysis, Supervision, Validation, Writing—review & editing. LO: Investigation, Supervision, Writing—review & editing. SL: Conceptualization, Funding acquisition, Methodology, Project administration, Resources, Supervision, Writing—review & editing.
