# Supplementary material for: Evaluation of recombinant baculovirus clearance during rAAV production in Sf9 cells using a newly developed fluorescent-TCID50 assay
Source: Front Med (Lausanne). 2024 Jan 22;11:1302648. doi: 10.3389/fmed.2024.1302648 (PMC10839039; doi:10.3389/fmed.2024.1302648)
Supplement: Supplementary file 1 [file Table_1.DOCX]

**Table S1. Summary of rBV removal and inactivation during AAV manufacturing**

| **Process** | **Type** | **LRV** |
| --- | --- | --- |
| Lysis (Sarkosyl and Triton X-100) | Inactivation | ≥4.3±0.2 |
| AAVx affinity chromatography | Removal | 5.4 |
| Low pH hold (pH3.0) | Inactivation | 2.9±0.4 |
| CsCl ultracentrifugation | Removal and Inactivation | 2.1 |
| NFR filtration | Removal | ≥5.1±0.3 |
| Total |  | ≥18.9 |
